# Beneficial Oral Biofilms as Smart Bioactive Interfaces

**DOI:** 10.3389/fmicb.2018.00107

**Published:** 2018-01-31

**Authors:** Beatrice Gutt, Qun Ren, Irmgard Hauser-Gerspach, Piotr Kardas, Stefan Stübinger, Monika Astasov-Frauenhoffer, Tuomas Waltimo

**Affiliations:** ^1^Laboratory for Biointerfaces, Swiss Federal Laboratories for Materials Science and Technology, St. Gallen, Switzerland; ^2^Department of Preventive Dentistry and Oral Microbiology, University Center for Dental Medicine, University of Basel, Basel, Switzerland; ^3^Hightech Research Center of Cranio-Maxillofacial Surgery, University of Basel, Basel, Switzerland

**Keywords:** biofilm, periodontitis, bacterial lysate, cell cytotoxicity, interface

## Abstract

Periodontitis is a very common health problem caused by formation of pathogenic bacterial biofilm that triggers inflammation resulting in either reversible gingivitis or irreversible periodontal hard and soft tissue damages, leading to loss of teeth when left untreated. Commensal bacteria play an important role in oral health in many aspects. Mainly by colonizing oral tissues, they (i) contribute to maturation of immune response, and (ii) foreclose attachment of pathobiont and, therefore, prevent from infection. The main goal of the study was to investigate if blocking of receptors on a commensal biofilm can prevent or reduce the attachment of pathogenic strains. To do so, biofilm produced by commensal *Streptococcus sanguinis* was treated with whole cell lysate of pathobionts *Fusobacterium nucleatum* or *Porphyromonas gingivalis*, followed by incubation with respective strain(s). The study revealed significant reduction in pathobiont adhesion to lysate-treated commensal biofilm. Therefore, adhesion of pathobionts onto the lysate-blocked biofilm was hindered; however, not completely eliminated supporting the idea that such approach in the oral cavity would benefit the production of a well-balanced and healthy bioactive interface.

## Introduction

Periodontitis begins as reversible gingivitis and can develop into irreversible periodontal soft and hard tissue destruction and if left untreated result in loss of teeth. Currently, both diseases are grouped into two categories according to the degree, severity and activity of the tissue destruction (Armitage, 1999; Lindhe et al., 2008).

The bacterial compositions in biofilms from a healthy periodontium are different from those in periodontitis (Newman and Listgarten, 1999; Wade, 2013; Lamont and Hajishengallis, 2015). Commensal microbiota plays an important role in maintaining oral health. The simple presence of such commensal community of bacteria in the mouth controls and inhibits colonization of possible pathogenic bacteria (Vollaard and Clasener, 1994; Wade, 2013). Commensal oral streptococci make the major proportion of early colonizers, composing up to 80% of adhered bacteria within the first 8 h after tooth cleaning (Diaz et al., 2006; Dige et al., 2009). These strains produce various adhesins, which allow them to bind numerous human cell and bacterial receptors. *Streptococcus sanguinis*, a frequently found commensal bacterium is able to encode more than 90 polypeptides, which are potentially mediate adhesion and create suitable conditions for adhesion of pathobionts (Xu et al., 2007). When balance within commensal biofilms is disrupted (e.g., pH change), pathobionts can adhere and accumulate on oral surfaces. Microbiota causing periodontitis is comprised of a mixed species community; however, it is dominated by different Gram-negative anaerobic bacteria (Mombelli and Décaillet, 2011). *Fusobacterium nucleatum* is known to co-aggregate with virtually all other bacteria acting as a bridging organism by binding to streptococci as well as to pathobionts, such as *Porphyromonas gingivalis*, *Treponema denticola*, *Prevotella intermedia*, and *Tannerella forsythia* (Kolenbrander et al., 2010).

The treatment of periodontitis is centered on the control of pathogenic biofilms (Mombelli and Décaillet, 2011) by improving oral hygiene or applying scaling and root planning that may be combined with antimicrobial treatments in certain clinical situations (Feres et al., 2015). However, disadvantages of such approach, like the possible resistance of bacteria in biofilms to the antimicrobial agents remain a concern. Such treatment is always an ecological intervention and improvement is gained by the suppression of periodontal pathobionts. Alternative approach has been reported, based on the inhibition of pathobionts like *P. gingivalis* that would help to reverse dysbiotic changes shifting the composition of the entire biofilm community toward commensal species (Hajishengallis, 2014; Lamont and Hajishengallis, 2015). Such approach might allow to control and influence biofilm formation. Current research data indicate that the range and mechanistic basis of such shift are not entirely understood; however, it is known that bacteria–bacteria and bacteria–host interactions are involved (Wright et al., 2013). In this study a novel concept was applied, namely using the whole cell lysate derived from the pathobionts *F. nucleatum* and *P. gingivalis* for blocking the receptors of commensal biofilms that can be found in the oral cavity.

**FIGURE 1:**
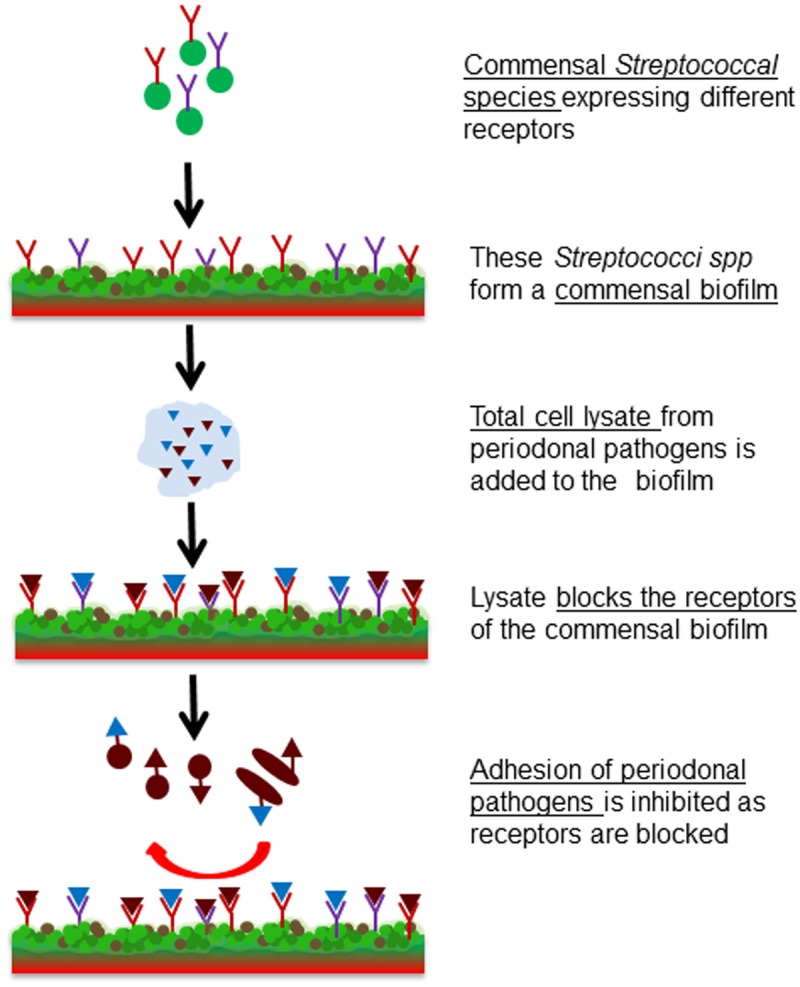
Schematic representation of the project idea. Formation of a commensal biofilm of *S. sanguinis* whose binding receptors are blocked by the bacterial cell lysate of *F. nucleatum* or *P. gingivalis*. Consequently pathogenic colonization is prevented.

The aim of this study was to investigate whether receptor blocking can be used to efficiently prevent pathobiont adhesion. For this, the early colonizer *S. sanguinis* was used to form a model commensal biofilm, whose adhesin-receptors were then blocked by the lysates of two pathobionts, to eliminate or reduce attachment or co-adhesion of pathogens (**Figure 1**). The commensal biofilm thus acts as a smart bioactive interface that helps to regulate the rate of attachment and multiplication of pathobionts. This system could be beneficial for controlling the pathogenesis of periodontitis.

## Materials and Methods

Chemicals and reagents were purchased from Sigma–Aldrich (Buchs, Switzerland) if not mentioned otherwise.

### Commensal Biofilm Formation

All bacteria strains were obtained from The Leibniz Institute DSMZ, German Collection of Microorganisms and Cell Cultures GmbH (Braunschweig, Germany).

*Streptococcus sanguinis* (DSM 20068) was grown in Schaedler bouillon (Becton Dickinson; Allschwil, Switzerland) at 37°C while shaking (160 rpm) for 16 h. The overnight culture was diluted to OD_600_ = 0.2 in fresh Schaedler bouillon supplemented with 0.1% sucrose and 100 μl culture was added per sample in 96-well microtiter plates (Falcon, Thermo Fisher Scientific, Reinach, Switzerland). Plates were incubated anaerobically at 37°C and 40 rpm for 24 h.

### Commensal Biofilm Blocking by Lysate and Adhesion of Secondary Species

*S. sanguinis* biofilms were washed with 0.9% NaCl and 100 μl of either periodontal pathobiont *F. nucleatum* (DSM 20482) or *P. gingivalis* (DSM 20709) lysates (100 μg/ml; preparation described below) was added to each well and incubated with the lysate for 15 min, 37°C. Wells incubated with 0.9% NaCl served as controls. Then the biofilms were washed twice with 0.9% NaCl and for secondary adhesion *S. sanguinis*, *F. nucleatum*, or *P. gingivalis* were added to the wells. Prior to addition, both pathobionts were grown for 72 h in thioglycollate enriched with hemin and vitamin K1, diluted to OD_600_ = 0.2 with fresh medium. 100 μl of this was added to the lysate-blocked *S. sanguinis* biofilm. The samples were then incubated for 24 h at 37°C. Thereafter the biofilms were washed twice with 0.9% NaCl and prepared either for crystal violet (CV) staining or fluorescence *in situ* hybridization (FISH).

### Bacterial Cell Lysate Preparation

Four Brucella blood agar plates supplemented with hemin and vitamin K1 with either *F. nucleatum* (DSM 20482; anaerobic incubation for 3 days) or *P. gingivalis* (DSM 20709; anaerobic incubation for 6 days) were used to prepare one batch of lysate. 1 ml lysis buffer (50 mM Tris-HCl pH 7.5, 200 mM NaCl, 5% glycerol, 1 mM 1,4-dithiothreitol) was added per plate; bacterial colonies were scratched off with a spatula (Heathrow Scientific, Vernon Hills, IL, United States). The suspension was collected to a 15 ml tube (TPP Techno Plastic Products AG, Trasadingen, Switzerland) and centrifuged for 10 min at 4°C at 7197 *g* (Centrifuge 5430R, Eppendorf AG, Hamburg, Germany). The supernatant was discarded and the weight of the pellet was determined. For each 100 mg of cell pellet 1 ml lysis buffer was added [supplemented with 10 μl 100 mM phenylmethane sulfonyl fluoride (PMSF)/ml of cell suspension]. This suspension was transferred to microtubes (Sarstedt AG & Co., Nümbrecht, Germany), already containing 300 mg of glass beads (Glass beads 425–600 μm, acid washed; Sigma–Aldrich, Buchs, Switzerland). Cells were disrupted by bead milling for 1 min (Scientific Industries; Disruptor genie, New York, NY, United States). Thereafter, lysozyme was added to a final concentration of 300 μg/ml and the suspension was incubated at 37°C for 30 min. After incubation a second bead-milling step was performed. Subsequently, the cell debris was removed by centrifugation for 30 min at 11300 *g* (MiniSpin^®^ plus, Eppendorf AG, Hamburg, Germany). Cell lysate was stored at -20°C. The concentration of cell lysate was determined by using Bradford assay and the reproducibility was confirmed by SDS silver staining (Thermo Scientific, Rockford, IL, United States).

### Biofilm Quantification by Crystal Violet Staining

**FIGURE 2:**
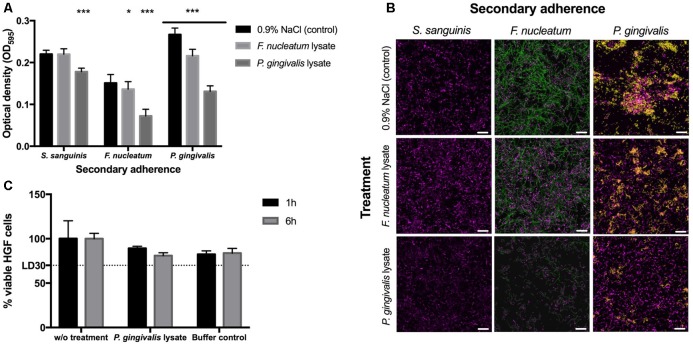
Biofilm formation and cytotoxicity effects. **(A)** Crystal violet staining after 48 h. The 24-h-old *S. sanguinis* biofilms blocked with lysate or 0.9% NaCl before secondary adherence of *S. sanguinis*, *F. nucleatum*, or *P. gingivalis* for another 24 h (*N* = 16). Asterisks denote statistical significance as follows: ^∗^*P* < 0.05, ^∗∗^*P* < 0.01, ^∗∗∗^*P* < 0.001 (*N* = 18). **(B)** FISH images to confirm the results obtained by crystal violet staining (*S. sanguinis* depicted in magenta, *F. nucleatum* in green, and *P. gingivalis* in yellow; scale bar: 10 μm) (*N* = 3). **(C)** Cytotoxic effects of *P. gingivalis* lysate on human gingival cells (HGCs). The lethal dose (LD) as indication of lethality of 30% of the cells is indicated as LD30 (*N* = 6). All data are presented as mean values ± standard deviation.

The crystal violet (CV) staining was performed as described by Stiefel et al. (2016). In detail, 250 μl of 0.5% crystal violet solution was added per sample and incubated for 30 min at room temperature. The wells were washed twice with 300 μl dH_2_O to remove surplus dye; additionally stain from upper borders of the wells was removed with a paper towel dipped in 70% ethanol. The wells were let to dry and 100 μl of 96% ethanol was added to each well to dissolve crystal violet. Biofilms were quantified by measuring OD_595_ (Synergy HT Multi-Detection Microplate Reader, BioTek^®^, Luzern, Switzerland). All data were analyzed using GraphPad Prism 6 (GraphPad Software, Inc., La Jolla, CA, United States). Analysis of the statistical differences between two samples was performed by one-way ANOVA and Tukey–Kramer’s *post hoc* test. The statistical significance is defined as follows: ^∗^*P* < 0.05, ^∗∗^*P* < 0.01, ^∗∗∗^*P* < 0.001 (**Figure 2A**).

### Biofilm Characterization by FISH and CLSM

Oligonucleotide DNA probes, labeled at the 5′-end with Cy3 and Cy5 or with 6-carboxyfluorescein (FAM) and additionally labeled at the 3′-end (Microsynth AG, Balgach, Switzerland), are listed with their sequences and specificities in Astasov-Frauenhoffer et al. (2012). Appropriate probe sequences for the specific detection of each bacterial strain in the biofilm have been described previously (Paster et al., 1998; Thurnheer et al., 2004; Guggenheim et al., 2009).

The samples were prepared as described in Astasov-Frauenhoffer et al. (2012). Briefly, the biofilms were fixed in 4% paraformaldehyde for 1 h at 4°C and washed with PBS. The samples were incubated for 15 min at 48°C in final hybridization buffer (0.9 mol/l NaCl, 20 mM/l Tris-HCl pH 7.5, 0.01% SDS) containing 30% formamide and then placed for 3 h at 48°C in the same solution with the oligonucleotide probes added (50 μg/ml for *S. sanguinis* and *P. gingivalis*, 150 μg/ml for *F. nucelatum*). After hybridization, the biofilms were immersed for 15 min at 48°C in washing buffer (102 mM/l NaCl, 20 mM/l Tris-HCl 7.5, 5 mM/l EDTA, 0.01% SDS) and thereafter washed twice with 0.9% NaCl. The samples were examined using a Zeiss point scanning confocal LSM700 Inverted microscope (Zeiss, Jena, Germany) fitted with three fixed lasers: 488, 555, and 639 nm. Filters were set to 500–530 nm for FAM, 570–610 nm for Cy3, and 650–730 nm for Cy5. The images were obtained using a 63x (numeric aperture 1.4) oil immersion objective, z-direction series were generated with the thickness of the slices set to 0.29 μm.

### Cytotoxicity of Bacterial Cell Lysates

Cytotoxic effects toward gingival fibroblasts [(HGF) human gingival fibroblasts; Catalog #2620, ScienCellTM, Carlsbad, CA, United States] of bacterial lysates and buffer were examined (ISO10993-5). HGF cells were seeded 1 day prior incubation with bacterial cell lysates with 15000 cells per well (200 μl) of a 96-well-microtiter plate to reach a confluence of 70%. Cells were incubated overnight at 37°C with 5% CO_2_. Hereafter, the HGF cells were treated with lysis buffer (50 mM Tris-HCl pH 7.5, 200 mM NaCl, 5% glycerol, 1 mM 1,4-dithiothreitol supplemented with PMSF and lysozyme) as control and bacterial cell lysates for 1 h and 6 h. Cells without treatment served as additional control. Cell viability was determined via MTT [3-(4,5-Dimethylthiazol-2-yl)-2,5-diphenyltetrazolium bromide, a tetrazole] assay by measuring the absorbance at 480 nm (Synergy HT Multi-Detection Microplate Reader, BioTek^®^, Luzern, Switzerland) to determine metabolic activity of the HGF cells.

## Results and Discussion

The main goal of this work was a proof-of-concept *in vitro* study to investigate whether using receptor-blocked commensal biofilms adhesion of pathogenic bacteria could be controlled. To validate this idea, a model commensal biofilm of the known early colonizer *S. sanguinis* was formed. In order to block binding receptors of the commensal biofilm and thus prevent the adhesion of pathobionts, bacterial cell lysates of known oral pathobionts such as *F. nucleatum* and *P. gingivalis* were applied. In a third step this blocked commensal biofilm was challenged with the same oral pathobionts to test the efficiency of the inhibition of adhesion rates. The designed system is illustrated in **Figure 1**.

*Streptococcus sanguinis* was chosen as a model organism to form a single-species biofilm due to its high presence in *in vivo* commensal biofilms. Commensal *Streptococci* spp. (*S. sanguinis*, *S. mitis*, *S. oralis*) are known for expressing multiple classes of adhesins for other species present in the oral cavity in later stages for biofilm formation. However, they are also known for maintaining a healthy oral microbial community, so that no periodontitis is triggered as long as they have the majority within the biofilm (Kreth et al., 2016). Once the commensal biofilm is challenged (e.g., due to changes in pH, presence of oxygen, changes in the flow of nutrition) periodontal pathobionts like *F. nucleatum* and *P. gingivalis* are typically found in the oral cavity (Jakubovics and Kolenbrander, 2010).

Crystal violet staining was used to measure the differences in biofilm content after lysate-blocked commensal biofilms and control biofilms were incubated in secondary adhesion step by *S. sanguinis*, *F. nucleatum*, and *P. gingivalis*, respectively (**Figure 2A**). Furthermore, FISH analysis was applied to study the distribution of individual species within the biofilms (**Figure 2B**). Results revealed that both lysates of the pathobionts blocked the receptors on the pre-existing commensal *S. sanguinis* biofilm and therefore reduced the adherence rate of *F. nucleatum* and *P. gingivalis*. Compared to the control without any cell lysate, lysate of *P. gingivalis* led to a 20% reduction of biofilm formation when the commensal *S. sanguinis* was added to the lysate blocked commensal biofilm; however, approximately 50% reduction was obtained when either of the pathobionts was used for secondary adhesion. These results led to a conclusion that lysates of *P. gingivalis* can efficiently block binding receptors of these particular commensal biofilms, which are essential for adhesion of other possible pathobionts. Nevertheless, it is important to note that the adhesion of further *Streptococcal* species was also slightly reduced.

Fluorescence *in situ* hybridization results confirm the CV staining data that treatment of commensal biofilms with *P. gingivalis* lysate prior incubation with *F. nucleatum* or *P. gingivalis* culture was found to decrease their ability to attach to the surface of the commensal biofilm (**Figure 2B**). Moreover, treatment of *S. sanguinis* biofilm with *P. gingivalis* cell lysate influenced only slightly the structure of the commensal biofilm. FISH results demonstrated that commensal biofilms blocked by *P. gingivalis* lysate prevented attachment of both pathobionts. At the same time the adhesion of new *S. sanguinis* cells was only slightly impaired, showing that the auto-aggregation is still sufficient to furthermore establish the stability within commensal biofilms and that the lysate blocking is more efficient against the pathobionts. This supports the main idea of this study that commensal biofilm is expected to hinder the adhesion of pathobionts and the resulting deleterious effects of pathogenic colonization. However, the purpose of the introduced commensal biofilm blocked by lysates is not to foster a complete elimination or decimation of oral bacteria or their development, but to produce a healthy and well-balanced environment, namely a biofilm, which can prevent a detrimental shift into an infectious state. Furthermore, our investigation adds new data on the ecological intervention in the oral cavity where adhesion of periodontal pathobionts is suppressed while recolonization of bacterial biofilms by host-compatible commensal species still takes place (Wade, 2013; Hajishengallis, 2014; Lamont and Hajishengallis, 2015).

Finally, *P. gingivalis* lysate as the most promising candidate was tested for its cytotoxic effects toward HGFs (**Figure 2C**). Compared to cells without treatment, bacterial cell lysates and the corresponding buffer controls all showed a reduction of 15–20% in HGF cell viability (**Figure 2C**). The mean of reduction caused by cell lysate correlates to the reduction caused by buffers itself. Thus, not the components of the obtained bacterial cell lysate, but the buffers used for the lysis caused this reduction. As cell viability was above 80% the bacterial cell lysates can be considered as not cytotoxic (**Figure 2C**).

## Conclusion and Perspectives

This study showed that pathobiont adhesion could clearly be inhibited when commensal biofilm formation is controlled and influenced, which allows creation of a smart bioactive interface acting as a safeguard against infection. However, due to the expression of various types of adhesins when main receptors are blocked, it is important to evaluate also more complex commensal biofilms and lysate mixtures, to validate the efficacy of the concept introduced here. As there are a variety of microbes found in the oral cavity, there is always more than one competitor strain present *in vivo*. Thus, both single-species bacterial cell lysate and a mixture of different bacterial cell lysates need to be prepared and could lead to a broader spectrum of blocking a commensal biofilm. The mixture of different bacterial cell lysates is most likely a superior solution in the prevention of outbalance of pathobionts in an established commensal biofilm *in vivo*. In addition for further studies it is necessary to evaluate further bacterial species most relevant for the development of commensal biofilms and the establishment of a reliable *in vitro* system culturing these bacteria under close to *in vivo* conditions. From a public health point of view, the use of such system could provide a novel alternative strategy for controlling periodontitis.

## Author Contributions

BG, PK, and MA-F designed the study, performed experiments and data analysis, interpreted the analyzed results, and coauthored the manuscript. QR, IH-G, SS, and TW designed the study, coordinated research and helped to author the manuscript. All authors contributed valuable advice on the analyzed results, read, and approved the final manuscript.

## Conflict of Interest Statement

The authors declare that the research was conducted in the absence of any commercial or financial relationships that could be construed as a potential conflict of interest.
